# Rhesus macaque and mouse models for down-selecting circumsporozoite protein based malaria vaccines differ significantly in immunogenicity and functional outcomes

**DOI:** 10.1186/s12936-017-1766-3

**Published:** 2017-03-13

**Authors:** Timothy W. Phares, Anthony D. May, Christopher J. Genito, Nathan A. Hoyt, Farhat A. Khan, Michael D. Porter, Margot DeBot, Norman C. Waters, Philippe Saudan, Sheetij Dutta

**Affiliations:** 1 Structural Vaccinology Laboratory, Malaria Vaccine Branch, Walter Reed Army Institute of Research, 503 Robert Grant Avenue, Silver Spring, MD 20910 USA; 2 Division of Veterinary Medicine, Walter Reed Army Institute of Research, 503 Robert Grant Avenue, Silver Spring, MD 20910 USA; 3 Malaria Vaccine Branch, Walter Reed Army Institute of Research, 503 Robert Grant Avenue, Silver Spring, MD 20910 USA; 4 Cytos Biotechnology, Wagistrasse 25, 8952 Schlieren, Switzerland

**Keywords:** Circumsporozoite protein, Vaccine, Mice, Rhesus, Macaque, Malaria

## Abstract

**Background:**

Non-human primates, such as the rhesus macaques, are the preferred model for down-selecting human malaria vaccine formulations, but the rhesus model is expensive and does not allow for direct efficacy testing of human malaria vaccines. Transgenic rodent parasites expressing genes of human *Plasmodium* are now routinely used for efficacy studies of human malaria vaccines. Mice have however rarely predicted success in human malaria trials and there is scepticism whether mouse studies alone are sufficient to move a vaccine candidate into the clinic.

**Methods:**

A comparison of immunogenicity, fine-specificity and functional activity of two Alum-adjuvanted *Plasmodium falciparum* circumsporozoite protein (CSP)-based vaccines was conducted in mouse and rhesus models. One vaccine was a soluble recombinant protein (CSP) and the other was the same CSP covalently conjugated to the Qβ phage particle (Qβ-CSP).

**Results:**

Mice showed different kinetics of antibody responses and different sensitivity to the NANP-repeat and N-terminal epitopes as compared to rhesus. While mice failed to discern differences between the protective efficacy of CSP versus Qβ-CSP vaccine following direct challenge with transgenic *Plasmodium berghei* parasites, rhesus serum from the Qβ-CSP-vaccinated animals induced higher in vivo sporozoite neutralization activity.

**Conclusions:**

Despite some immunologic parallels between models, these data demonstrate that differences between the immune responses induced in the two models risk conflicting decisions regarding potential vaccine utility in humans. In combination with historical observations, the data presented here suggest that although murine models may be useful for some purposes, non-human primate models may be more likely to predict the human response to investigational vaccines.

## Background

Malaria remains a major health concern in the tropics despite control efforts using available drugs and insecticides. There is a broad consensus that a highly effective vaccine is needed. Two vaccines developed by Sanaria^®^ (PfSPZ) and GlaxoSmithKline Vaccines (RTS,S), have demonstrated the proof-of-concept that a vaccine can protect against controlled human malaria infection (CHMI) [1]. Both PfSPZ and RTS,S vaccines induce potent immune responses against *Plasmodium* sporozoites, in particular against the circumsporozoite protein (CSP). Efforts are underway to improve upon the success of PfSPZ and RTS,S, but there is considerable debate on what animal model should be used as the critical path for advancing novel vaccine candidates to human trials. The rodent model for malaria is based on *Plasmodium berghei*, *Plasmodium chabaudi* or *Plasmodium yoelii* parasites that naturally infect African thicket rats [2]. These parasites have been adapted to grow in mouse strains for routine laboratory experiments and provide easy access to blood and liver stages. Rodent models have led to successful translation of many malaria drugs, however the down-selection of vaccines using mice has had mixed benefits. While the irradiated sporozoite vaccine can protect both rodents and humans [3], many sub-unit vaccines have failed to translate protection from mice to humans. A CSP DNA vaccine can induce very potent sterilizing protection against *P. berghei* and *P. yoelii* in mice [4, 5], but *Pf*CSP-based DNA vaccines did not protect humans [6]. Likewise, the *P. yoelii* merozoite surface protein-1 (MSP1) [7] and *P. chabaudi* apical membrane antigen-1 (AMA1) [8] candidates have been known to protect mice, but human-use formulations of *Pf*MSP1 [9] and *Pf*AMA1 [10] vaccines confer limited protection in humans.

A lack of translation of observations from mice to humans is not surprising given that the common ancestor of humans and mice lived ~75 million years ago and significant differences in the invasion biology of rodent and human malaria parasites exist [11]. Rhesus macaques (*Macaca mulatta*) and humans share a more recent common ancestor that lived approximately 25 million years ago [12], and several malaria parasites that naturally infect rhesus, such as *Plasmodium knowlesi* and *Plasmodium cynomolgi*, are human transmissible [13]. Rhesus challenge models can be used to evaluate vaccines that inhibit parasite growth in both the liver and blood, whereas human trials with such vaccines mandate drug treatment immediately at the onset of blood-stage patency [14]. *P. cynomolgi* parasite challenge in rhesus allows cross-species protection studies to be conducted on *Plasmodium vivax* vaccines [15], circumventing the need for a much more complex controlled *P. vivax* human challenge. Rhesus models have also been useful in testing novel concepts such as multivalent and whole parasite vaccines [16, 17]. There are several instances where the rhesus model has predicted the success of human malaria vaccine trials. The rationale for selection of the AS01 over AS02 adjuvant for the RTS,S vaccine was based on a rhesus trial [18] that was subsequently corroborated by CHMI and in the field [19, 20]. In another study, the adenovirus serotype-35 CSP prime and RTS,S boost improved the IFN-γ^+^ T cell responses in rhesus, but also showed lower antibody titres than RTS,S alone [21]. These findings were closely replicated in humans with improved T cell responses and reduced antibodies in the prime-boost arm [22]. In addition, the irradiated *P. knowlesi* sporozoite vaccine protected rhesus against virulent *P. knowlesi* challenge and CD8^+^ T cell responses correlated with protection [23]; this has been replicated in CHMI model where irradiated *Plasmodium falciparum* sporozoite vaccine elicited protection was also characterized by induction of CD8^+^ T cell responses [3, 24, 25]. The rhesus model has not only predicted the success but also the failure of many experimental malaria vaccines. Formulations of *Pf*MSP1 and *Pf*AMA1 that induced low level in vitro merozoite growth inhibition activity in the rhesus model [26] failed to protect humans [9, 10]. Soluble *Pv*CSP that induced relatively low repeat specific antibodies in rhesus [27] also failed to confer sterile protection against *Pv*CHMI [28]. These studies provide direct evidence that rhesus can accurately predict the immunogenicity and protection outcomes of experimental human malaria vaccines.

Despite excellent predictive capability, the scarcity and cost associated with non-human primates remain problematic and although rhesus malaria parasites are human transmissible, both *P. falciparum* and *P. vivax* cannot infect rhesus and hence direct challenge experiments are not feasible. A way to overcome the species barrier is to develop transgenic parasites. While transgenic rhesus parasites have been difficult to produce and select, transgenic rodent parasites carrying *P. falciparum* and *P. vivax* antigen genes are now routinely used to answer biological questions [29, 30]. Mouse-human chimeric parasites expressing *Pf*CSP, *Pfs*25, *Pf*MSP1, and *Pv*CSP have been used to compare the efficacy of human *P. falciparum* and *P. vivax* vaccines in mice [31–41]. In particular, transgenic parasites have been very useful to study the role of *Pf*CSP during hepatocyte invasion [42, 43] and challenge models have been optimized to down-select *Pf*CSP vaccines using *P. berghei* parasites that carry functional *Pf*CSP genes [44, 45]. Considering previous examples where mouse data have not translated to protection in humans, there remains an uncertainty in the validity of using a mouse model for down-selecting *P. falciparum* and *P. vivax* malaria vaccines prior to CHMI. To address this issue, immunological responses to two vaccines were compared here in mice and rhesus.

The vaccines tested were derived from a nearly full-length *Pf*CSP construct expressed in *Escherichia coli* that contains the N-terminal region, 19 NANP, and three NVDP repeats followed by the C-terminal region. Also contained within the protein sequence were two inter-species conserved motifs of *Pf*CSP: ‘region I’ (located just before the central repeats) and the cysteine-rich ‘region II’ (located after the repeats). Both of these conserved regions have been implicated in hepatocyte invasion [42, 46]. In order to produce a comparator particulate vaccine, the soluble CS protein was chemically coupled to recombinant Qβ capsid protein. Qβ protein self-assembles into 25 nm virus-like particles within *E. coli* cytosol and has been used as vaccine carrier in many human trials [47–50]. During assembly in the cytosol, *E. coli* RNA is encapsulated inside the Qβ virus-like particles and this bacterial RNA serves as a potent adjuvant since it is a strong TLR7/8 agonist [51, 52]. It was recently reported that soluble CSP chemically conjugated to Qβ particles (Qβ-CSP) induced superior immunogenicity and efficacy compared to soluble CSP in several adjuvants in mice [53]. In the current study, the Qβ-CSP and soluble CSP vaccines were adjuvanted in Alum and used to immunize mice and rhesus. Although human data on these vaccine formulations are not available, rhesus responses were treated as a close surrogate for human responses based on their proven predictive value in previous malaria vaccine trials. Qβ-CSP generally induced higher overall antibody responses than CSP in both mice and rhesus, but differences in epitope specificity, avidity and functionality of antibodies observed between the two species argue that rhesus ought to remain the choice model for the final down-selection step before advancing to human trials.

## Methods

### Vaccine antigens

CSP expression and purification has been reported previously [45]. The conjugation of CSP to Qβ phage particle was carried out using a cysteine-based cross-linker and has been described earlier [53]. The CSP content of the Qβ-CSP vaccine was determined as the sum of conjugated and unconjugated CSP to ensure the CSP content was equal in all comparator vaccine groups. Method of quantifying CSP in Qβ-CSP has been previously described [53].

### Animals

Six- to eight-weeks old female C57Bl/6 mice (*Mus musculus*) supplied by Jackson Laboratory (Bar Harbor, ME, USA) were housed at the Walter Reed Army Institute of Research (WRAIR) animal facility for the duration of the study (IACUC-approved protocol number 11-MVD-15). The room was maintained at 21–23 °C, with a relative humidity of 3–70%, 1–15 air changes hourly and a 12:12-h light:dark cycle. At the time of this study, all mice were negative for Ectromelia virus, Mouse Cytomegalovirus (MCMV), Mouse Parvovirus (MPV), Murine Norovirus (MNV), Mouse Adenovirus (MAdV-1/MAdV-2), Polyomavirus, Lymphocytic Choriomeningitis Virus (LCMV), Sendai virus, Pneumonia Virus of Mice (PVM), Reovirus 1,2, and 3, Rotavirus, Mouse Hepatitis Virus (MHV), Theiler’s Murine Encephalomyelitis Virus, Mycoplasma pulmonis, CAR bacillus, Clostridium piliforme (Tyzzer’s Disease), and Helicobacter. Mice were singly housed in suspended, polycarbonate shoebox-type cages with filter tops on Beta Chip bedding Q3 (Northeastern Products Corp, Warrensburg, NY, USA), and each cage had shreddable Q4 nesting material (Nestlets, Ancare, Bellmore, NY, USA) and an Igloo or similar tube for enrichment. Mice were fed a Q4 pelleted rodent food (RMH3000, Lab Diet, St Louis, MO, USA) and provided drinking water ad libitum. Sentinel mice used to monitor the health status of the experimental animals tested negative for all monitored pathogens.

Adult male rhesus macaques of Indian origin (*M. mulatta*), 9–15 years old and 6–15 kg weight were housed at the WRAIR animal facility and used under an IACUC-approved protocol, number 13-MVD-12L. Monkeys used were malaria naïve as assessed by ELISA against CSP antigen, but were used in other non-malaria studies in the past. The environment was maintained at 20–22 °C, with a relative humidity of 30–70%, 10–15 air changes hourly and a 12:12-h light:dark cycle. All animals were free from overt clinical signs of illness, deemed to be in good health and tested negative for Macacine herpesvirus 1, measles, Simian Retrovirus, Simian Immunodeficiency Virus and Simian T-cell Leukemia Virus, and tuberculin skin test. Animals were singly housed, fed a commercial diet (Lab Diet 5038, Purina Mills International, Brentwood, MO, USA) and provided water ad libitum. Environmental enrichment was provided in accordance with WRAIR Veterinary Service Programs standing operating procedures.

### Mouse vaccination, bleed and challenge

To determine optimal group size, ELISA titres following three doses of a different CSP-based vaccine were log transformed and the standard deviation fed into the Russ Lenth online power calculator using the 2-sample *t* test algorithm [54]. The above calculation showed that n = 7 per group would have 80% power to discern ~two-fold differences in mean titre. Mice (n = 7 per group) received three doses of 2.5 µg CSP at 3-week intervals as soluble protein or Qβ-CSP formulated in Alum (300 µg/ml aluminum content; Alhydrogel; Brenntag Biosector, Frederikssund, Denmark). Alum was mixed 1:1 v/v with the antigen by medium vortexing for 3 s every 5 min for 30 min and 100 µl of this vaccine was administered via 50 µl intra-muscular injection into each of the thigh muscles. The antigen dose and vaccine schedule were the same as has been previously used to differentiate between formulations as it gives sub-saturating levels of protection against this specific transgenic parasite strain [44, 45, 53]. Mice were bled at 3 weeks after each immunization, except for the final bleed that was collected at 2 weeks after the last dose. To measure protective efficacy, all mice were challenged with 3000 transgenic *P. berghei* sporozoites expressing a functional copy of the *Pf*CSP gene at 2 weeks after the last dose, as described previously [42, 44]. Briefly, sporozoites were collected using the Ozaki method [55] and injected into the lateral tail vein of mice. Mice were monitored daily and parasitaemia was detected using thin blood smears beginning at day 4 post-challenge. Blood smears were fixed and stained with Giemsa. Mice found to be parasitaemic for 2 consecutive days were sacrificed and recorded as ‘not protected’ while mice that did not develop blood stage parasitaemia up to 14 days post-challenge were reported as ‘protected’.

### Rhesus vaccination and bleeds

Optimal group size was determined as above and n = 5 was shown to have 98% power to discern a two-fold difference in mean titre. Rhesus monkeys (n = 5 per group) were anaesthetized and the thigh area was shaved. A total 0.5 ml of the vaccine was administered intra-muscularly in the outer thigh muscle. Each vaccination contained 25 µg CSP or Qβ-CSP in Alum (300 µg/ml aluminum content). The schedule for vaccination was at 0, 1 and 3 months. Rhesus were bled 2 weeks after each vaccination for serology. The antigen dose for rhesus was chosen based on the proposed human dose of this vaccine, which is ten-fold higher than that used in the mouse model. The vaccine schedule was based on a previous publication on a rhesus monkey trial with RTS,S and an adenovirus-based CSP vaccine [21].

### ELISA

ELISA plates (Immulon 2HB) were coated overnight at 4 °C with either the full-length CSP antigen (FL, 50 ng/well), the repeat peptide ((NANP)_6_C peptide at 20 ng/well) or a recombinant protein encoding the C-terminal region of CSP (50 ng/well) essentially as described previously [44]. The secondary antibody used for the mouse and rhesus ELISA were HRP-conjugated anti-mouse or anti-rhesus IgG, respectively (Southern Biotech, Birmingham, AL, USA). Horse Radish Peroxidase labelled secondary antibodies against Rhesus IgG1 (clone 7H11), IgG2 (clone 3C10) and IgG3 (2G11) were obtained from the NIH Non-human Primate Reagent Resource. The antibody titre was calculated as the serum dilution that produced an absorbance of 1.0 optical density (OD) units using Gen5™ software (Biotek). To measure avidity, the above ELISA protocol was modified with an additional 6 M urea incubation step for 10 min following the primary antibody incubation. Equal volume of phosphate buffered saline (PBS) was added for a control. Avidity index was defined as the titre (OD = 1.0) in the presence of urea divided by the titre in the PBS control. Epitope specific ELISA was done using the following peptides—NT: DNAGTNLYNELEMNYYGKQENWYS; RI + repeat: KLKQPADGNPDPNANPNVDPNANPNVDPNANPNVDPNANP and RII: WSPCSVTCGNGIQVRIKPGSANKPKDELDYANDIEKKICKMEKCSS. All peptides were coated at 200 ng/well and 1:100 serum dilution was added to the well for 1 h. The reaction was developed as above and OD at 415 nm was recorded.

### Competitive ELISA

Monoclonal antibodies 2A10 and 5D5 were obtained from the Malaria Research and Reference Reagent Resource Center MR4 (BEI Resources, Manassas, VA, USA). To determine the ability of polyclonal serum to block the binding of CSP-specific monoclonal antibodies (mAb), a competitive ELISA was developed. In brief, ELISA plates were coated overnight at 4 °C with FL antigen (15 ng/well) and then washed with PBS + Tween. Non-specific binding was blocked with PBS + 1% albumin for 1 h at room temperature (RT) and then washed. Seventy-five microliter/well of serially diluted (two-fold) mouse or rhesus serum was added to the plate and incubated for 1 h at RT. The mAbs were labelled with HRP using Lightning-Link HRP conjugation kit (Novus Biologicals, Littleton, CO, USA). Seventy-five microliter (~15 ng) of HRP-conjugated N-terminal mAb 5D5 [56], C-terminal mAb 2F12 [53] or NANP mAb 2A10 [57] was then added directly to the wells and incubated for 1 h at RT. Plates were then washed and developed by the addition of 50 µl/well of ABTS peroxidase substrate system (KPL) for 1 h at RT and stopped by adding 50 µl of 5% SDS. Absorbance was read at 415 nm on a microplate reader (Synergy 4, Biotek) using Gen5™ software (Biotek).

### Sporozoite neutralization assay

To assess in vivo protective efficacy of rhesus antibodies in mice, a sporozoite neutralization assay (SNA) was developed. In brief, 50 µl of untreated serum from individual rhesus monkeys was incubated for 10 min with 50 µl of RPMI containing 3000 transgenic *P. berghei* sporozoites. Mice were then challenged intravenously with 100 µl of the serum/sporozoites mixture. Pre-immune rhesus serum from each of the test monkeys was used as a control. After challenge, mice were monitored for parasitaemia as described above.

### Statistical analysis

Graphs show individual data points and/or the mean ± SEM (geometric mean for graphs representing titres) of each group. In all cases, a *P* value of <0.05 was considered significant, as determined by an unpaired two-tailed *t* test comparing the soluble and particulate platforms to each other within the species. Statistically significant difference in group means was indicated as * (P < 0.05), ** (P < 0.01), *** (P < 0.001) or **** (P < 0.0001). Graphs were plotted and statistics were assessed by using GraphPad Prism software (La Jolla, CA, USA).

## Results

### Qβ-CSP analysis and vaccination

**Fig. 1:**
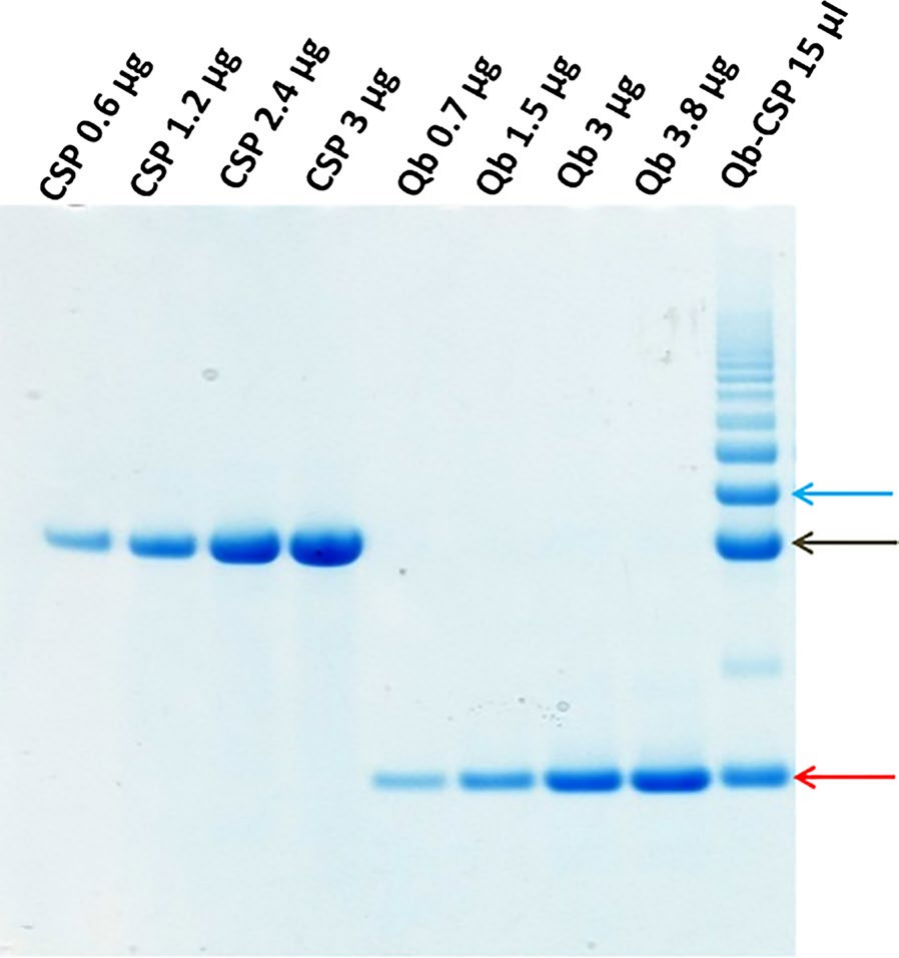
Purity of CSP, Qβ protein and conjugate Qβ-CSP. A range of CSP (*black arrow*, ~45 kDa) and Qβ protein (*red arrow*, ~15 kDa) amounts were run on reducing SDS-PAGE; loaded amounts are noted above each well. Conjugated Qβ-CSP (15 µl) was loaded in the last lane. Gel stained with Coomassie blue. Bands migrating at ~55 kDa and above (*blue arrow*) correspond to Qβ-CSP conjugate(s)

Qβ-CSP vaccine was prepared as previously described [53] and analysed by SDS-PAGE. Figure 1 shows that CSP and the Qβ proteins were highly pure and both migrated as a single band on reducing SDS-PAGE. Following conjugation of CSP and Qβ, multiple bands were detectable (Qβ-CSP lane). A ~45 kDa band corresponded to free CSP (black arrow), ~15 kDa band corresponded to the Qβ monomer (red arrow) and ~55 kDa band (blue arrow) corresponded to the conjugated Qβ-CSP molecule. Bands above 55 kDa in the Qβ-CSP lane corresponded to a CSP molecule linked to Qβ multimers that were generated during the cysteine-coupling reaction. Densitometric quantification of conjugation revealed that 19% of Qβ monomers had a CSP molecule conjugated to them. Hence, 34 CSP molecules were conjugated to an assembled Qβ particle that is composed of 180 Qβ monomers [58]. To be able to compare the two vaccines, identical amounts of CSP were administered. For the Qβ-CSP vaccine conjugated and free CSP in the preparation were quantified as previously described [53]. The animal studies were based on previously published reports of CSP vaccine selection in mice [45] and in rhesus [21]. Mice received three vaccinations of 2.5 µg CSP at week 0, 3 and 6 and rhesus received three vaccinations of 25 µg CSP (expected human dose) at month 0, 1 and 3.

### Kinetics of antibody response

**Fig. 2:**
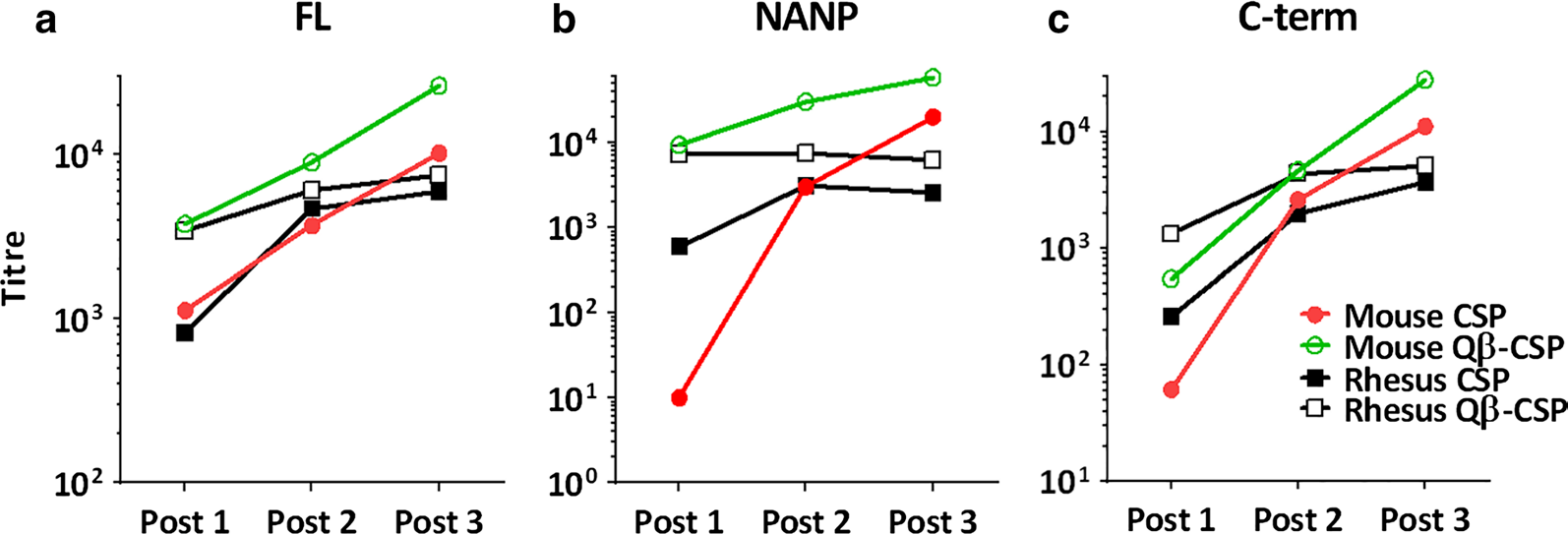
Immunogenicity of CSP and Qβ-CSP in mice and rhesus. ELISA results against (**a**) FL antigen, (**b**) NANP repeat or (**c**) C-terminus for serum collected 2–3 weeks after each immunization with CSP (*solid symbols*) or Qβ-CSP (*open symbols*). Plotted are group geometric mean antibody titre for mice (*coloured lines*, *red* or *green*, n = 7) and rhesus (*black lines*, n = 5). Fold increase of Qβ-CSP relative to CSP at each time-point is shown in Table 1

Comparing the trend of antibody titres over three immunizations, mice (Fig. 2, coloured lines) showed a clearer separation between the Qβ-CSP (green) and CSP (red) vaccine induced FL, NANP and C-term titres as compared to rhesus (black lines). In both models Qβ-CSP showed higher responses than CSP after the first dose, however, mice and rhesus differed substantially in the response to booster vaccines. In mice, successive doses of Qβ-CSP and CSP boosted titres but little or no boosting was observed beyond the second dose in rhesus. Indeed, no boosting of NANP titres were observed in rhesus after the first dose of Qβ-CSP, while in mice boosting post-second and post-third was observed.

Both models agreed that FL, NANP and C-terminus titres were higher for the Qβ-CSP vaccine than CSP following the first vaccination (Table 1), but the magnitude of the difference between vaccines was maximal for the NANP epitope in mice. The NANP titre for the Qβ-CSP was >900-fold higher than CSP in mice, but only 12-fold higher in rhesus (Table 1). In mice, the Qβ-CSP maintained a clear immunological edge over CSP after the second dose with statistically significant differences in FL and NANP titres. In contrast, the differences between the two vaccines after the second dose were less clear in rhesus (Table 1). The immunogenicity difference between the two vaccines was further reduced in both models after the third dose, but mice continued to show significantly higher FL and NANP response in the Qβ-CSP group. In rhesus, the NANP titre of the Qβ-CSP vaccine group was ~2.4-fold higher than CSP, however this difference was not statistically significant.

**Table 1:** Relative enhancement of CSP titres by conjugation to Qβ

	Mouse			Rhesus		
	(Qβ-CSP: CSP titre ratio)			(Qβ-CSP: CSP titre ratio)		
	FL	NANP	C-term	FL	NANP	C-term
Post 1	3.3**	930****	8.8**	4.1	12*	5.1*
Post 2	2.4**	10***	1.8	1.2	2.4	2.2*
Post 3	2.5*	2.9*	2.5	1.3	2.4	1.4

CSP titre ratios of group geometric mean antibody titre for mice (n = 7) and rhesus (n = 5) 2–3 weeks after each immunization. ELISA was run against FL, NANP, and C-terminus plate antigens. Significant differences between log-transformed CSP and Qβ-CSP titres at each time point for each plate antigen was determined by two-tailed *t* test, denoted by * (P < 0.05), ** (P < 0.01), *** (P < 0.001) or **** (P < 0.0001)

### Avidity and subtypes

**Fig. 3:**
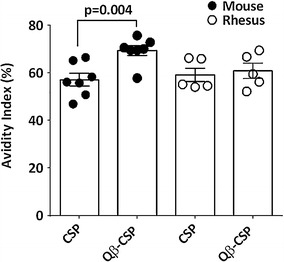
Serum antibody avidity against FL antigen. Avidity index for mice (*solid points*, n = 7) and rhesus (*open points*, n = 5) was calculated as the titre (OD = 1.0) in the presence of urea as a percentage of the titre in the PBS control. *Bars* represent mean avidity index ± SEM against FL antigen at 2 weeks post-third vaccination. *P* values are shown for statistically significant differences between CSP and Qβ-CSP determined by two-tailed *t* test

Avidity and IgG subtypes were measured against FL antigen post third immunization. In mice, Qβ-CSP generated significantly higher avidity antibodies relative to CSP, while no avidity differences between the two vaccines were detectable in rhesus after the third dose (Fig. 3). As was previously reported [53], higher levels of IgG2c were induced by the Qβ-CSP vaccine compared to the CSP vaccine (data not shown); however, attempts to measure sub-classes in rhesus using either rhesus-specific or human-specific secondary antibodies were unsuccessful as only the IgG1 antibody (rhesus or human) reacted with rhesus serum.

### Region-specific bias

**Fig. 4:**
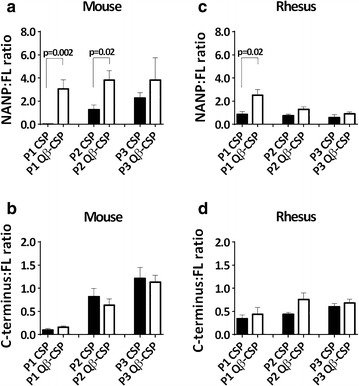
Relative immunogenicity of the NANP repeat and C-terminus regions of CSP. Data are expressed as the mean titre ratio (*bars*) ± SEM (*whiskers*) for mice (**a**, **b**; n = 7) and rhesus (**c**, **d**; n = 5) of NANP repeat titres (**a**, **c**) or C-terminus titres (**b**, **d**) to their respective FL antigen ELISA titre at post-first (P1), post-second (P2) and post-third (P3) vaccination with either CSP (*solid bars*) or Qβ-CSP (*open bars*). *P* values are shown for statistically significant differences between CSP and Qβ-CSP determined by two-tailed *t* tests

Difference in antibody bias towards specific regions of the vaccine molecule was determined by normalizing individual NANP and C-term titres by the respective FL titre. The NANP bias of Qβ-CSP antibodies was greater than CSP antibodies (NANP:FL titre ratio >2) following the first and second vaccination in mice (Fig. 4a). Even after the third dose, a notable NANP bias was observed for Qβ-CSP in mice, although this was not statistically significant compared to CSP. The higher level of NANP bias induced by Qβ-CSP was replicated in rhesus (NANP:FL titre ratio ~2) (Fig. 4c), albeit only after the first dose. Mice and rhesus displayed no difference in C-terminus bias between the two vaccines (Fig. 4b, d).

### Epitope-specificity

**Fig. 5:**
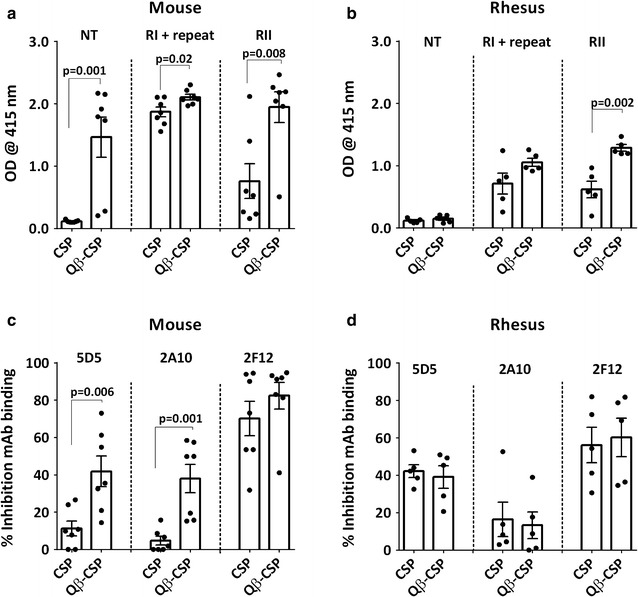
Region- and epitope-specific responses. **a**, **b** Points represent optical density from a peptide ELISA at 1:100 serum dilution. Peptides comprised of the N-terminus (NT), conserved region I plus NVDPNANP repeats region (RI + repeat), and the cysteine rich region II of C-terminus (RII). **c**, **d** Points represent per cent inhibition of HRP-labelled 5D5 (N-terminus), 2A10 (NANP repeat) or 2F12 (C-terminus) mAb in the presence of individual sera. Inhibition is expressed as the percent decrease in optical density obtained by ELISA with mAb alone. *Bars* represent group means and* whiskers* represent standard error of the mean (SEM) for mouse (n = 5) and rhesus (n = 7) serum from 2 weeks post-third vaccination. *P* values are shown for statistically significant differences between CSP and Qβ-CSP determined by two-tailed *t* tests

To assess the relative abundance of antibodies against CSP epitopes, post-third vaccination sera were analysed by a direct peptide ELISA and a monoclonal antibody competition ELISA. The ‘NT’ peptide corresponded to a sequence in the N-terminus, the ‘RI + repeat’ peptide was based on the conserved region-I and NVDPNANP repeat sequence, and the ‘RII’ peptide corresponded to the cysteine-rich region-II within the C-terminus. Mouse sera raised against Qβ-CSP vaccine contained higher levels of NT peptide-reacting antibodies than CSP vaccine sera; in contrast, the corresponding rhesus sera showed little NT peptide reactivity (Fig. 5a, b). Mice also showed a higher R1 + repeat and RII peptide response in favour of Qβ-CSP vaccine after the third dose (Fig. 5a, b), while only the RII peptide ELISA showed a significant difference between the two vaccines in rhesus. The ability of post-third mouse and rhesus serum to inhibit binding of mAbs to the FL antigen was also tested in a competition ELISA (Fig. 5c, d). Mouse antibodies showed that the Qβ-CSP vaccine induced higher N-terminus (mAb 5D5) and NANP repeat (mAb 2A10)-specific binding inhibition relative to the CSP vaccine (Fig. 5c). Rhesus antibodies, however, failed to discern any difference between the two vaccines using this competition ELISA (Fig. 5d). Increased mAb 5D5 inhibition (Fig. 5c) by Qβ-CSP mouse sera correlated with the higher NT peptide ELISA responses (Fig. 5a). Overall, more epitope-specific differences between Qβ-CSP versus soluble CSP were seen in mice than in rhesus.

### Protection

**Fig. 6:**
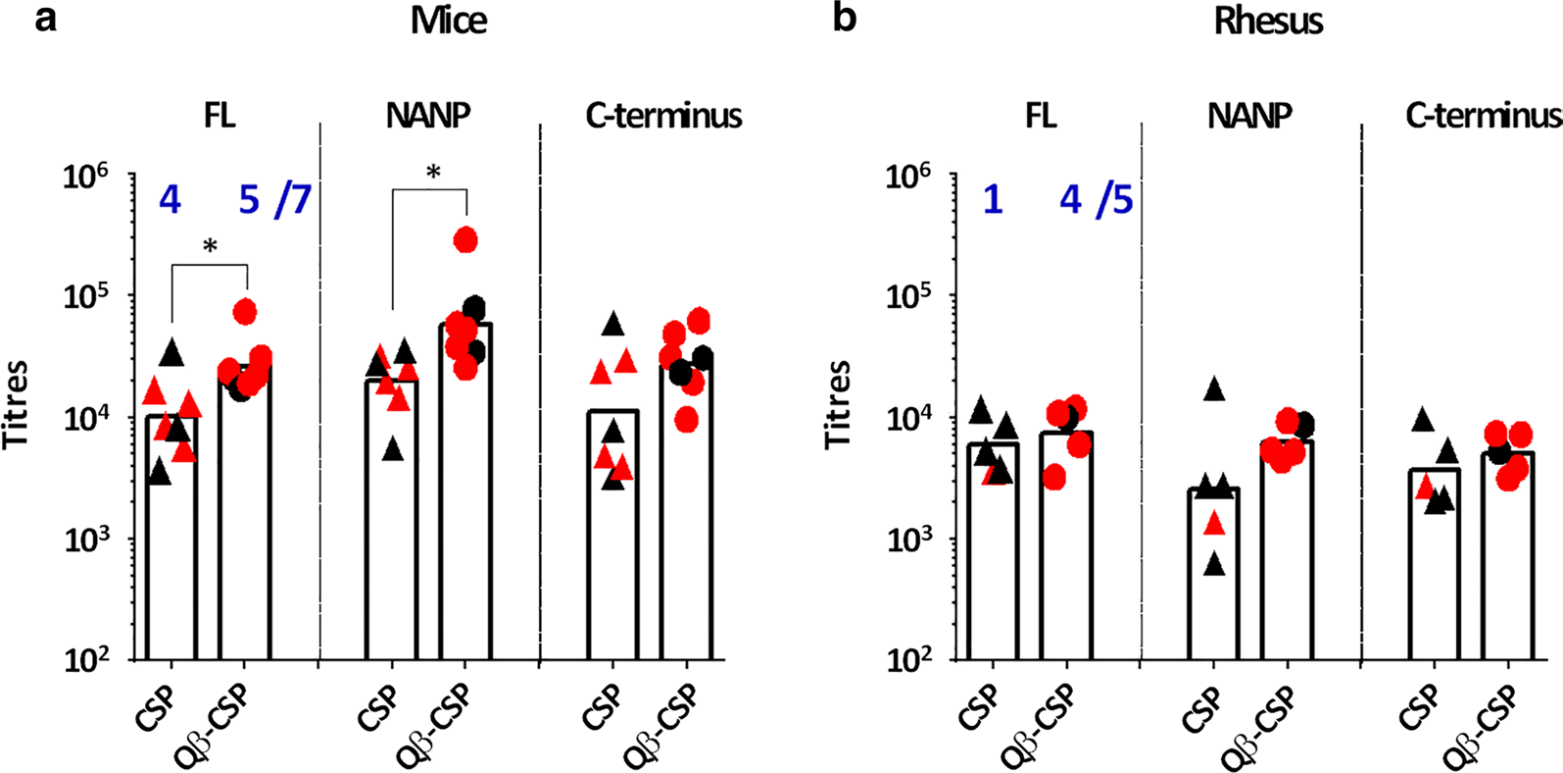
Protection efficacy of CSP versus Qβ-CSP vaccination. Individual titres for mice (n = 7) and rhesus (n = 5) 2 weeks post-third vaccination. *Red symbols* represent (**a**) mice immunized and protected in direct challenge with 3000 transgenic *P. berghei* sporozoites (Tr-Pb) or (**b**) mice protected by challenge with 3000 Tr-Pb in SNA with rhesus serum. *Blue numbers* represent the number of protected mice out of the total. *Bars* represent group geometric mean. *P* values are shown for statistically significant differences between CSP and Qβ-CSP determined by two-tailed *t* tests

To evaluate the protective efficacy of the CSP and Qβ-CSP vaccines in mice, a transgenic *P. berghei* sporozoite line that expressed the full-length CSP gene of *P. falciparum* was used to infect the mice 2 weeks after the third vaccine dose. All control mice became infected by day 7 and there was no significant difference in the level of protection induced by the CSP and Qβ-CSP vaccines (4/7 versus 5/7; Fig. 6a), even though the FL and NANP titres of the two groups were significantly different. Since no transgenic parasites for rhesus challenge were available, individual rhesus sera were tested using an in vivo SNA in the mouse transgenic parasite challenge model. All mice representing individual rhesus pre-immune sera were infected by day 5, but rhesus sera from 4/5 Qβ-CSP group monkeys showed protection while only 1/5 rhesus sera in the CSP group conferred protection (Fig. 6b). The higher protection in the Qβ-CSP group SNA was loosely associated with a higher titre (although not significant) and smaller spread of NANP repeat antibodies (Fig. 6b).

## Discussion

A comparison of two malaria vaccines was conducted to determine if mice and rhesus models provided congruent antibody read-outs. While there were differences in vaccine schedule and vaccine dose between species, the two models agreed on several immunologic outcomes: (1) Qβ-CSP vaccine had overall higher immunogenicity; (2) within the CSP molecule, the relative immunogenicity of the repeat NANP epitope was specifically improved by particulate presentation on Qβ; and, (3) Qβ-CSP was better at priming antibody responses, but soluble CSP was able to boost antibody levels after subsequent doses, particularly against the NANP repeat. Apart from the similarities, the mouse and rhesus models differed from each other in the magnitude, kinetics, epitope breath and functionality of antibodies, summarized in Table 2. In general, more immunological differences between Qβ-CSP and CSP vaccines were detected in mice. The rhesus model revealed a key difference in functional activity of antibodies induced by the two vaccines using a SNA while mice failed to discern this difference upon direct challenge.

**Table 2:** Differences between mouse and rhesus models

Parameter	Mouse model	Rhesus model
Priming vaccination (Table 1; Fig. 2)	~900-fold higher Qβ-CSP NANP titres after first dose	~12-fold difference between vaccines
Booster vaccination (Fig. 2)	Titres were boosted by each vaccination	No boosting beyond second dose
Immunogenicity (Table 1; Figs. 2, 6)	Significantly higher FL and NANP titres for Qβ-CSP group after the third dose	Differences between vaccines was lower and not significant
Serum antibody avidity (Fig. 3)	Higher avidity of Qβ-CSP group	No difference in avidity
NANP epitope bias (Fig. 4)	NANP-biased Qβ-CSP group response after each dose	NANP-biased response of Qβ-CSP after 1st dose only
NANP mAb competition (Fig. 5)	Higher mAb 2A10 inhibition by anti-Qβ-CSP	No difference in mAb inhibition detected
N-terminal response (Fig. 5)	Higher N-terminal response by Qβ-CSP	No difference in N-terminal response
Functional assay (Fig. 6)	No difference in protection observed after direct challenge	Qβ-CSP protected more mice in a neutralization assay

Summary of observed differences between animal models in the present study, with respect to comparison between Qβ-CSP and CSP vaccines. Tables and Figures illustrating noted differences are referenced for each parameter

Mouse models are being used at an increasing rate to down-select second-generation malaria vaccines, but it would be costly to ignore preceding investigations where mouse data did not necessarily translate to humans. To the authors’ knowledge, this is the first report where identical malaria vaccine formulations have been compared in C57BL/6 mice and rhesus, both of which are commonly used models in malaria vaccine development. The main difference between the CSP and Qβ-CSP vaccines was the enhanced immunogenicity of Qβ-CSP, which could have resulted from its particulate nature and/or the TLR7/8 agonist activity of the entrapped RNA [59, 60]. Others have also reported that *Pf*CSP antibodies can be readily boosted by a third immunization in mice while no additional boosting beyond the second vaccination was observed in rhesus [18, 26, 34, 61]. Not surprisingly, the boosting pattern of RTS,S in humans is similar to the rhesus after the second dose [62]. Overall, compared to rhesus, mice greatly amplified the immunogenicity differences between soluble CSP and Qβ-CSP, and this may reflect differences in the innate immune response pathways of the two species as was observed using a CpG oligodeoxynucleotide adjuvant for a *Leishmania* vaccine [63]. Furthermore, monkeys harbour much more genetic and epigenetic variability stemming from differences in individual age, microbiome and physiology, while the impact of these factors on immunogenicity is largely suppressed in inbred mouse strains.

Despite the relatively low immunogenicity of soluble CSP in Alum, mice were protected in a direct challenge experiment. While reasons remain unclear, high-level protection with Alum-adjuvanted soluble CSP has been reported previously in mice [53], but it is also known that Alum-adjuvanted CSP vaccines do not induce significant protection in humans [64–67]. A similar observation has been made in influenza where several Alum-adjuvanted vaccines that induce protective levels of haemagglutinin response in mice do not protect humans [49, 68]. Using a functional SNA, rhesus revealed that Qβ-CSP + Alum was more protective than CSP + Alum. This functional activity was loosely associated with the magnitude and spread of NANP titres that also happen to be the best correlate of protection for CSP-based malaria vaccines in CHMI [69]. A recent report has suggested that antibodies against the N-terminus of CSP may offer additional protection, as they can block a proteolytic processing step during sporozoite invasion [56]. In mice, the Qβ-CSP induced higher N-terminal responses than CSP, but there was no difference in rhesus. These data highlight a fundamental problem with down-selecting human vaccines in mice and cautions against over-interpreting mouse vaccine data that show enhanced epitope-specific responses, enhanced avidity or even enhanced protection, as these results may not translate to higher mammalian species. Rhesus on the other hand are not ideal for mechanistic studies in immunology as attempts to sub-class rhesus macaque IgG using available rhesus (NIH NHP Reagent Resource) or human-specific reagents were unsuccessful. Others have reported similar limitations of using human sub-class reagents to analyse rhesus IgG [70]. Rhesus produces three distinct IgG sub-classes, but there is a much greater diversity among rhesus Ig genes compared to humans [71] and the functional equivalency of rhesus and known human IgG sub-classes has not been established.

While it would be ideal to use rhesus as they best emulate the human host, mice continue to be the pre-eminent in vivo model for malaria vaccine work as evidenced by more than 1900 Pubmed hits compared to 148 on rhesus malaria vaccine research. It was found that the mouse immune system can amplify small immunological differences between formulations. Mice are therefore useful for early decision-making where one formulation must be picked among many. It is important however that the selection criteria in mice be kept fairly stringent, such as using a sterile protection outcome and conservative statistical tests to minimize Type-1 error. In rhesus, a less obvious difference between formulations was seen. Careful balance between sample size and study cost is therefore necessary to reduce the risk of a Type-2 error while down-selecting vaccines in rhesus. These data and past limitations of mouse models suggest that non-human primate immunogenicity in combination with functional assays needs to be a preferred model for final down-selection prior to clinical trials.

## Conclusions

This report has direct implications for numerous ongoing efforts to down-select CSP-based malaria vaccine candidates and cautions against the use of only murine models for final down-selection of potential vaccine candidates for human studies. The immunogenicity of two malaria vaccine candidates in mice and rhesus were compared and it was found that, while the two models shared some of the immunologic outcomes, there were key differences between them. Mice showed a clear immunologic superiority of a particulate Qβ-CSP formulation over soluble CSP, while the difference in responses to the soluble and particulate antigens was muted in rhesus. With the caveat that these vaccines have not yet been tested in humans, this study suggests that there are limitations of reliance on data derived solely from the mouse model in predicting the human response to investigational vaccines and underlines the likely superiority of the rhesus model in this context.
